# Promoting Tissue Repair by Micrograft Stem Cells Delivery

**DOI:** 10.1155/2020/2195318

**Published:** 2020-01-28

**Authors:** Letizia Trovato, Fabio Naro, Francesco D'Aiuto, Federico Moreno

**Affiliations:** ^1^University of Turin, Turin, Italy; ^2^University of Sapienza, Rome, Italy; ^3^UCL Eastman Dental Institute, London, UK

Stem cell therapy provides new insights for the treatment of diseases or disorders that cannot yet be successfully managed through conventional care. Current cell-based approaches are mainly focused on the use of Mesenchymal Stem Cells (MSCs) which are multipotent stem cells with unique biological properties and are readily available from almost every organ and tissue. MSCs are becoming widely used to improve outcomes for multiple clinical scenarios: heart failure, wound healing, bone regeneration, and many others. Despite promising in vitro and in vivo results, the need for extensive manipulation steps to obtain sufficient cell numbers for delivery significantly increases the regulatory, practical, and financial barriers for the routine use of these novel therapies. Helping to overcome some of these limitations, the use of micrografts represents a suitable approach for tissue regeneration. Micrografting can be used to deliver not only progenitor cells but also growth factors and matrix extracellular components naturally available in the tissues which further increase the regenerative potential.

Only few clinical trials have reported the efficacy of stem cell therapy; further research must be conducted to investigate also the safety of these techniques in the long term. Micrografting is attracting a growing interest from the research community since it involves easy/quick and simple procedures which reduce the amounts of donor tissues required while reducing morbidity.

In this special issue, new procedures able to promote repair or regeneration in areas such as plastic surgery and wound healing are reported, using both stem cells and micrografts. In the manuscript by M. M. Bashir et al., the authors evaluate the effect of adipose tissue grafts mixed with adipose tissue-derived stem cells (ASCs) to promote fat graft retention in patients with contour deformities of the face showing an improvement in clinical outcomes when compared to the control group. The papers from M. Riccio et al. and M. M. Tresoldi et al. report the results from two clinical trials investigating the use of dermal micrografts in the treatment of traumatic or complex wounds in different patient populations. M. Riccio et al. present the management of 70 patients affected by traumatic wounds of the lower and upper limbs characterized by an extensive loss of substance. The results showed complete wound healing after 35 to 84 days. On the other hand, M. M. Tresoldi et al. treated elderly patients with wither micrografts or another dermal skin substitute, aiming to compare the efficacy for wound repair of both tissue replacement therapies. All patients reached a good degree of reepithelialization, but the authors failed to demonstrate a statistically significant difference between both groups. Finally, the study from A. Andreone et al. proposes a new approach for the management of burns based on the combination of platelet-rich fibrin and micrografts as a spray-on skin, reporting a rapid reepithelialization and impressive resurfacing.

This special issue also introduces the reader to fine original basic research focused on the biological and cellular mechanisms involved in tissue repair in different conditions, helping to better elucidate some of the key players activated in these processes. The study conducted by C. Zhang et al. assessed the role of autophagy in cavernosal endothelial dysfunction of diabetic rats, explaining the therapeutic effect of urine-derived stem cells (USCs) which upregulated autophagic activity in the cavernosal endothelium, ameliorating cavernosal endothelial dysfunction and improving erectile dysfunction. Another interesting study carried out by F. Wang et al. explored the potential of injectable hydrogels as cell-carrying scaffolds able to mimic the condition of the natural extracellular matrix (ECM) of nucleus pulposus (NP), providing binding sites for cells. The authors reported that, in rats, the transplantation of injectable hydrogel-loaded NP-derived MSCs can delay the level of intervertebral disc (IVD) degeneration while promoting its regeneration and restoring the structure and ECM content of degenerated NP eight weeks after treatment.

Skeletal muscle has a remarkable capacity to regenerate following injury due to the presence within the adult muscle of a tissue-resident stem cell population known as satellite cells (SC). These cells remain quiescent until physical exercise, or muscle damage induces their activation. In the manuscript by S. Aguanno et al., the researchers present a culture model of the myogenic C2C12 cell line in suspension, able to self-assemble in three-dimensional cultures and to form a system able to preserve the quiescence and the staminality of these cells. The findings from this study support the conclusion that this model could be used to investigate the pathways controlling SC quiescence entrance and maintenance.

Micrograft therapies are a promising and affordable alternative to improve skin regeneration through enhancement of the endogenous wound repair processes. In the publication from M. Balli et al., the authors provide new insights which might contribute to a better understanding of the molecular pathways involved in micrograft-induced wound repair. Remarkably, the authors identified new key players in the wound healing process such as the active protein-1 (AP-1) and member Fos-related antigen-1 (Fra-1) which enhanced cell migration in mouse adult fibroblasts and human keratinocytes treated with soluble. These results were confirmed when inhibition of both ERK and AP-1 completely reverted the in vitro wound closure otherwise induced by the soluble micrograft treatment. This outcome highlights the important role of ERK-dependent transcriptional activity of AP-1 in the promotion of micrograft-induced wound closure.

We sincerely hope that the articles published in this special issue can help researchers to better understand the potential roles of stem cell therapy and micrografting techniques for tissue regeneration, including the different advantages and disadvantages of both approaches. Further, the basic research manuscripts included in the issue provide new insights that improve the current understanding of the cellular and molecular mechanisms involved in the wound healing process, especially in pathological conditions such as diabetes or intervertebral disc degeneration.

